# The impact of petrol and diesel oil taxes in EU member states on CO_2_ emissions from passenger cars

**DOI:** 10.1038/s41598-023-50456-y

**Published:** 2024-01-02

**Authors:** Michał Ptak, Jarosław Neneman, Sylwia Roszkowska

**Affiliations:** 1 Department of Economics and Research on Development, Faculty of Economics and Finance, Wroclaw University of Economics and Business, Komandorska 118/120, 53-345 Wrocław, Poland; 2 Department of Institutional Economics and Microeconomics, Faculty of Economics and Sociology, University of Lodz, POW 3/5, 90-255 Lodz, Poland; 3 Institute of Economics, Finance and Management, Faculty of Management and Social Communication, Jagiellonian University, Lojasiewicza 4, 30-348 Kraków, Poland

**Keywords:** Environmental economics, Energy and society, Environmental economics, Environmental impact

## Abstract

The article aims to explain road CO_2_ emissions, including passenger car emissions in the EU member states, with the rates of indirect taxes (except VAT) for petrol and diesel oil. Apart from tax rates, the analysis includes some selected variables concerning economies and transport infrastructure, which impact CO_2_ car emissions. Compared to the existing literature, we focus on emissions from passenger cars and analyse more countries over a more extended period using more updated data. Our findings confirm that fuel taxes have a generally negative but limited impact on emissions from passenger cars. This impact is independent of whether we relate emissions to the number of inhabitants or GDP and is generally stronger in EU member states with higher taxes. In many countries, the economic affordability of fuels has significantly increased over the last few years. This phenomenon is another argument for a more active tax policy, i.e., general adjustment of the tax rates in line with inflation. There is also great importance for those adjustments in times of high fuel prices when governments are under tremendous pressure not only to stop tax increases but to reduce them, which was the case in 2022 after the Russian aggression on Ukraine.

## Introduction

In the European Union, passenger road transport depends on non-renewable fuels to a great extent, and most passenger cars are petrol- or diesel-powered^1–3^. In 2019, energy which was produced from these fuels constituted 38.8% and 52.6%, respectively, of the total energy resulting from the combustion of fuel used in road passenger transport^4^.

The combustion of fossil fuels in passenger cars generates significant externalities by emitting carbon dioxide and, in this way, contributes to climate change. In 2019, CO_2_ emissions from fuel combustion in passenger cars in the EU accounted for 18% of total carbon dioxide emissions from total fuel consumption and 61% of CO_2_ emissions from fuel consumption in road transport^4^.

In order to reduce carbon dioxide emissions from passenger cars, taxes levied on transportation fuels can be used to play the role of the Pigouvian taxes. Because CO_2_ emissions from vehicles are directly related to fuel use, fuel taxation is considered an "adequate" or "well-suited" instrument to internalise climate-related externalities generated by road transport vehicles^5,6^. Other negative external effects of transport are usually not directly proportional to fuel use; hence, fuel taxes would not be perfect instruments to internalise external costs related to noise or congestion^7^.

Taxes levied on motor fuels are consumption taxes that establish price signals providing incentives to reduce fuel consumption or improve the fuel efficiency of driving behaviour^5,8^. Drivers can reduce fuel use by adopting a more fuel-saving driving style^9^: keeping the proper tyre pressure^10^, driving shorter distances^11^, or choosing public transport^12^. In the long run, drivers can be encouraged to purchase more fuel-efficient or low-CO_2_ vehicles^13,14^ or move closer to their workplace^14^. Furthermore, reductions or exemptions stipulated in fuel taxes can stimulate increased consumption of biofuels. Of course, consumers ultimately react to final fuel prices (or changes in the prices) as fuel tax rates are components of the prices^5,15^.

Most petrol and diesel taxes in European Union member states have not been originally implemented for environmental reasons and thus are not optimally designed to reduce emissions, e.g. in terms of rate differentiation^6,16^. Taxes are often primarily revenue-raising tools^1^. Thanks to relatively high rates and large quantities of consumed fuels, taxes levied on transportation fuels have the potential to generate significant revenues which can be used to finance government expenditures, the costs of road construction and maintenance projects^17,18^ or improvements in public transport^12^.

In European Union countries, petrol and diesel oil are subject to indirect taxes, which are *ad quantum* taxes and *ad valorem* taxes. The former taxes are based on the unit (for example 1000 l) of the given type of fuel, and the latter (like VAT) are based on the price of the fuel^19^. VAT taxes are generally deductible for companies and affect relative prices differently than other indirect taxes^20^. For private car users, VAT is not deductible, but VAT does not play the role of a Pigou tax here. The 'only' purpose of VAT is to finance budgetary expenditures^21^. Therefore, VAT taxes levied on fuels and other environmentally harmful goods are often not considered environmental taxes.

Indirect taxes (other than VAT) levied on motor fuels in the EU are regulated by Directive 2003/96/EC (*Council Directive 2003/96/EC of 27 October 2003 restructuring the Community framework for the taxation of energy products and electricity*, 2003). The Directive sets minimum levels of taxation applicable to energy in the member states (359 euros per 1000 L of petrol and 330 euros per 1000 L of diesel oil). It permits the states to apply for specific reductions or exemptions. The directive's provisions relate to various fuel taxes, mainly traditional excise duties and carbon taxes. The latter are taxes based on the carbon content of fuels and are specially designed to reduce CO_2_-related externalities^22^. Carbon taxes together with CO_2_ emissions trading schemes impose in a direct (explicit) way carbon prices on emissions. Less directly, carbon prices are also created by traditional excise taxes on energy which are not levied in proportion to the carbon content of the fuel. These are often called “implicit carbon prices”^23^. Furthermore, energy tax rates can be converted into “implicit carbon taxes” using CO_2_ emission factors^5^.

In the commonly accepted classification of environmental taxes, it is assumed that taxes on petrol, diesel oil and other transport fuels are included in energy taxes. This group of taxes also includes taxes on fuels used outside transport (i.e. heating fuels) and taxes on electricity^24^. Transport taxes, which constitute a separate group of environmental taxes, can also contribute to the limiting of CO_2_ vehicle emissions. In the European Union, the role of these taxes in the limitation of greenhouse gas emissions can be considerable because the structure of vehicle acquisition (registration) or ownership taxes often takes CO_2_ emission or fuel consumption into account^25^. The Eurostat also includes pollution and resource taxes in the environmental taxes (sometimes also called environmentally related taxes)^24^.

This paper aims to explain passenger cars and road transport CO_2_ emissions in the European Union countries with the rates of indirect taxes (apart from VAT) on petrol and diesel oil. Knowledge of the effects of taxes is essential because it can define the significance of tax instruments for achieving the climate policy's national and international objectives^26^. Also, knowledge about the potential consequences of taxes allows the appropriate building of these taxes. Learning about the potential consequences of fuel taxes can ultimately limit social resistance to these instruments^27^. Energy taxes are perceived by the public opinion primarily as fiscal measures, while their environmental effectiveness is underestimated^28^.

The paper is organised as follows. Section "Taxes on petrol and diesel oil and CO2 emissions from passenger cars in the European Union" briefly describes the evolution of petrol and diesel oil taxes in the European Union countries from 2010 to 2019. In section "Related literature", we review estimates of the emission impacts of energy taxes. Section "Data" describes the dataset used for estimation. Section "Methodology, results and discussion" provides the results of the estimation of petrol and diesel oil taxes on CO_2_ emissions. Finally, section "Conclusion and policy implications" concludes and provides policy implications.

## Taxes on petrol and diesel oil and CO_2_ emissions from passenger cars in the European Union

**Figure 1:**
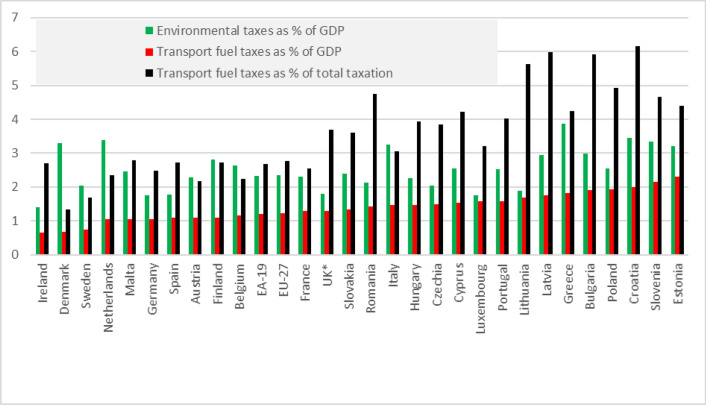
The share of environmental taxes and transport fuel taxes in GDP and total taxation in 2019. *2017. *Source*: European Commission and Directorate-General for Taxation and Customs Union, 2021.

In the European Union, petrol and diesel oil are the most heavily taxed fuels. Taxes levied on motor fuels are often higher than taxes levied on other transportation fuels and on heating fuels (considering the energy and carbon content of fuels)^5,7^. As can be seen from Fig. 1, taxes on transportation fuels constitute the majority of environmental taxes in some countries (e.g. Lithuania and the UK). In contrast, in others (e.g. Denmark, the Netherlands, Finland), they constitute less than a half of environmental taxes. The relative significance of transportation fuel taxes (measured in relation to GDP and total taxation) is higher in poorer countries of the EU.

**Figure 2:**
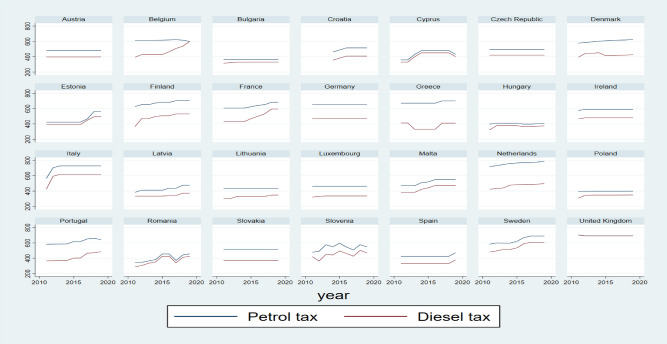
Tax rates applied to petrol and diesel oil in the European Union countries (euro per 1000 L, 2010–2019). *Source*: own calculations based on European Commission and International Energy Agency data.

**Figure 3:**
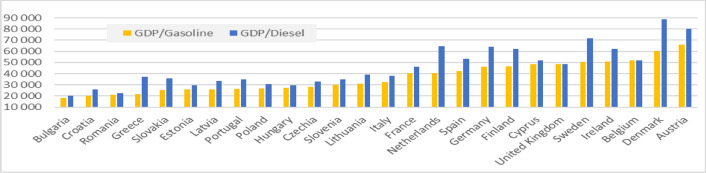
Economic affordability of excise on fuels (GDP per capita/tax rate per l of fuel, 2019). *Source*: own calculations based on European Commission and Eurostat data.

More prosperous EU countries generally have higher taxes (see Fig. 2). On the other hand, these countries can “afford” higher taxes, and the relative burden of these taxes is much lower due to the higher income of inhabitants. Suppose we relate the nominal GDP per capita to the tax rate per litre (expressed in current euros). In that case, we get a measure of the economic affordability of fuel taxes expressed in litres of petrol and diesel. In other words, this is the number of litres an average citizen can buy with GDP assuming that the price of petrol equals the tax rate. As seen from Fig. 3, the economic affordability of fuels defined in such a way is much lower in poorer EU member states. Actual differences are not that huge as the pre-tax price of the fuels is quite similar in all countries and usually constitutes more than a half of the price. During the high energy prices after the Russian invasion of Ukraine, public awareness of high prices pressured politicians to lower taxation. Unfortunately, both from the environmental and budgetary perspectives, the politicians were very eager to lower fuel taxes in many countries.

In EU member states, fuel taxes differ considerably (Fig. 2). In 2019, the rates of petrol taxes in some countries (the Netherlands, Italy, Finland, and Greece) were about twice higher than the minimum levels of taxation. Nevertheless, in such countries as Bulgaria, Hungary or Poland, petrol taxes only slightly exceeded the minimal levels of taxation.

Significant differences in tax rates among countries give rise to differences in final fuel prices^29^. Such differences between neighbouring countries create a possibility of fuel tourism. Cross-border fuel price differences can affect not only tax revenues but also the car fleet composition in one of the countries^30^.

In some EU member states (Germany, for example), petrol and diesel oil are subject to one indirect (excise) tax other than VAT. In such countries as Denmark, Finland, France, Ireland, Portugal, Slovenia and Sweden, fuels were taxed in 2019 with an "ordinary" energy tax and a carbon tax^24^. Other levies imposed on petrol or diesel oil in EU member states include, for example, strategic stockpile fees (Finland, Slovenia) and charges that generate revenues used for road maintenance (Poland, Portugal) . The price of motor fuels also includes value added taxes. In 2019, VAT tax rates applied to petrol and diesel oil ranged from 17% (Luxembourg) to 27% (Hungary)^31^.

Compared to diesel oil used for commercial uses and other fossil-based fuels, tax reliefs that can be applied to petrol and diesel oil used in passenger cars are very modest. Thus, the environmental effects of the taxes levied on fuels used in passenger cars in EU member states are not too negatively affected. As can be concluded from Fig. 2, petrol and oil tax rates in 2010–2019 considerably changed in some European Union states. As a result, petrol and diesel oil tax levels in particular countries in 2019 were usually higher than those in 2010. A particularly steep rise (at least 20%) in these taxes can be observed in the case of Cyprus, Greece, Estonia, Italy, Lithuania and Latvia. In some countries (Finland and France), only the rates of diesel oil tax rose significantly^31^.

Tax rates applied to petrol and diesel oil were changed relatively frequently in most EU member states. In a few countries, there was only one increase in these rates, and in three countries (Czechia, Germany and Slovakia), the rates of petrol and diesel taxes remained the same in the analysed period. Nevertheless, fuel tax rates in these countries in 2019 were close to the average European level. The petrol tax rate in Germany belonged to the highest ones in Europe^31^.

In the initial years of the analysed period, tax rates in some countries were raised due to the end of the transition periods for the adjustment of tax rates to the minimal levels described in 2003/96 directive. The adjustment of tax rates to the EU requirements was particularly acute in Cyprus and Greece^31^.

In some countries, (at least some) fuel tax rates were more or less regularly adjusted (Fig. 2). Yet in Poland, just the fuel charge, which is only a tiny part of the whole taxation level of petrol and oil, was adjusted^31^.

From 2010 to 2019, new taxes and payments were introduced in some countries. These new taxes were, among others, carbon taxes introduced in France (in 2014) and Portugal (in 2015). In Slovenia in July 2019, a carbon tax, which after 1997 was imposed on other fuels, was levied on petrol and diesel oil^32^.

The carbon tax case in France shows that fuel taxes can meet some opposition, and the design of the taxes can be affected by interest groups^18,33^. The carbon tax was introduced at 7 euros per tonne of CO_2_. The government intended to gradually increase the rate to 100 euros in 2030^34^. However, rising energy taxes, along with rising oil prices, led to mass protests in 2018. As a result, motor fuel tax rate increases were stopped in France and have remained the same since 2018^35^.

**Figure 4:**
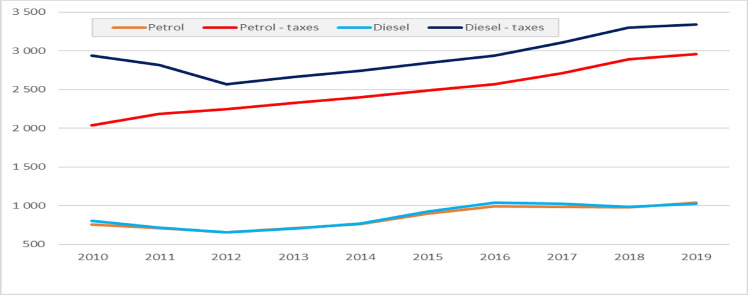
Economic affordability of fuels in Poland between 2010 and 2019, expressed as the number of litres of fuels or fuel taxes that could be purchased monthly with the average income. *Source*: Own calculations based on European Commission, Statistics Poland and UNECE data.

If fuel taxes are to reduce fuel consumption, then they should be adjusted to inflation or better to nominal disposable income or average income. However, as shown in Fig. 2, that differed in many UE countries. As a result, fuels' economic affordability increased significantly in most countries. Figure 4 shows the economic affordability of petrol and diesel in Poland between 2010 and 2019 concerning fuel taxes and the actual average price of fuels. That fact enables us to distinguish between changes in the economic affordability of fuels due to taxation and overall affordability, considering both tax and the pre-tax prices of fuels. In the case of petrol, in 2010, a person with an average income could buy monthly ca. 753 L of petrol and 803 L of diesel. In 2019, these values were 1036 and 1024 L; i.e., they went up by 38% and 28%, respectively. When fuel taxes are taken into account, then in 2010, an average income could “buy” fuel taxes in 2039 L of petrol and 2941 L of diesel. In 2010, these values were 2956 L and 3342 L; i.e., they went up by 45% and 14%, respectively. It implies that the fuel tax policy was very passive in Poland. A similar situation also took place in some other European countries; e.g., between 2010 and 2019, gross monthly wages went up by 28% in Germany, 36% in Slovakia and 43% in Czechia, whereas excise taxes on fuels were kept constant^31^.

It can be concluded from Fig. 2 that in the period of 2010–2019, the rates of tax on diesel oil increased in most countries more than the rates on petrol (see also^5^). As a result, the disproportion between the taxation of petrol and diesel oil (expressed in the country currency per 1000 L of a given type of fuel) decreased in 20 countries^31^. Diminishing differences in the rate of petrol and diesel oil taxes were supposed to reduce air pollution (diesel cars emit fine particulates and gas pollutants, which cause local air pollution)^7,36^. In Belgium and France, fuel taxation changes were motivated in this way^37^.

Lower taxes levied on diesel oil (on a volume basis) than on petrol are not justified from the point of view of climate policy, as the combustion of a litre of diesel emits more CO_2_ than that of petrol^7,36^. The favourable treatment of diesel has quite a long tradition in the EU. Such a way of levying fuel taxes resulted from the willingness to protect the competitiveness of commercial transport which uses diesel oil^5,7^ and the willingness to reduce the dependence on oil thanks to the higher fuel efficiency of diesel-powered cars. Similarly, the 2003/96/EC directive regulations assume lower tax rates for diesel oil, providing incentives for higher diesel consumption^7,24^. The abolition of the tax differentiation for petrol and diesel oil was provided for in the 2021 council directive restructuring the Union framework for the taxation of energy products and electricity^24^.

The only country where petrol and diesel oil tax rates have been identical for many years is Great Britain. In Belgium, tax rates for petrol and oil became even only in 2019^31^. Changes in fuel taxation were supposed to influence the changes in the structure of fleet in Belgium as of 2015^37^.

A non-optimal from the environmental point of view way of fuel taxation in the majority of EU member states^7^ resulted in *dieselisation*, i.e., the gradual replacement of petrol-powered vehicles with diesel ones^36^. Favourable tax treatment of diesel oil also caused an increase in CO_2_ emissions from passenger cars due to the rebound effect^47^. In transport, this phenomenon occurs when improved fuel efficiency reduces the cost of driving and increases the kilometres travelled. The increase in energy consumption due to higher demand for driving more fuel-efficient vehicles offsets (at least partially) the energy savings due to higher fuel efficiency^26,60^. For reviews of the rebound effect in transport, see^35^.

**Figure 5:**
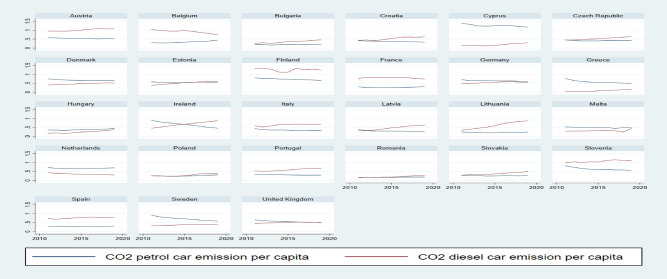
CO_2_ emissions from petrol and diesel passenger cars in tonnes per capita, 2010–2019. Source: Eurostat and UNFCCC secretariat data.

The described changes in transportation fuel taxation from 2010 through 2019 in the EU member states could have impacted transport's carbon dioxide emission level. Total CO_2_ emissions from the combustion of petrol and diesel oil in passenger cars in 28 EU member states rose slightly (by 2%) in the 2010–2019 years. In this period, emissions from petrol cars decreased by 13%. The fall in these emissions occurred in most EU member states. In some countries (Ireland, Greece, Latvia), emissions from petrol passenger cars were significantly limited (by over 40%). The situation was different for emissions produced from diesel oil combustion, which rose by 16%^4^. In 2010–2019 years, CO_2_ emissions generated by diesel passenger cars increased in 24 UE countries, see Fig. 5. To a large extent, this is due to the *dieselisation* of passenger cars in many UE countries. One can observe the substitution of petrol emissions for diesel emissions.

Inventory reports of some EU member states point to the fact that changes in taxes on road transport fuels or changes in prices of these fuels had an impact on fuel consumption in road transport and carbon dioxide emissions^38,39^. Other factors which influence the consumption of fuels and CO_2_ emissions include (inter alia):

- tax exemptions applied to transport biofuels^38^ and regulations requiring the use of biofuels in transportation^40^,
- biofuel consumption^41^ ,
- the general economic situation^39–41^,
- transportation activities^42^,
- the number of vehicles^39^ and their fuel efficiency^40^,
- the number of passengers using public transport,
- emission performance standards for new passenger cars^38^,
- vehicle taxes (fees) and subsidies on purchase (e.g. registration) and possession (e.g. circulation),
- economic affordability of cars and fuels, which is a function of taxes and subsidies, price of crude oil and disposable income,
- social attitude and awareness.

Some of these factors are interconnected, also with taxes on transportation fuels.

In this paper, we focus on emissions from passenger cars. Our approach is novel, as total emissions from road transportation are usually analysed in the EU. We found only one paper on the impact of the petrol tax on CO_2_ emissions from passenger cars^43^.

## Related literature

To date, several studies have estimated the effectiveness of fuel taxes on climate policy. In existing studies, the impact of taxes on CO_2_, GHG emissions, or their growth rate is often analysed. In many studies, the dependent variable is emissions per capita. Some of the studies are based on estimates of price elasticities and use emission factors of fuels to identify the impact of taxes on emissions^44^.

Several authors only focus on transportation fuel taxes^22,27, 44^. Studies also estimate the environmental impact of various "green" taxes. When this approach is used, the impact on total CO_2_ emissions is analysed, not just emissions from transport. It should be noted that some of the studies use fuel or oil prices (and alternatively taxes) as determinants of fuel consumption or CO_2_ emissions^3,8, 22, 27, 44–46^. González et al. (2019) use the change of petrol to diesel oil price ratio and other variables related to *dieselisation*^47^.

Studies estimating the environmental effectiveness of fuel taxes can be done either ex-ante or ex-post. Ex-ante evaluations investigate the effects of taxes to be introduced in a given country or the effects of hypothetical taxes, particularly carbon taxes^48^. In ex-ante evaluations, different tax rates are sometimes analysed^48^. Ex-post evaluations examine the environmental effects of existing taxes and sometimes use counterfactual simulations^22, 27, 45,49^. The authors use different methods to estimate the emission impact of environmental taxes. The methods include, for example, regressions, synthetic control methods, and general equilibrium models.

Variables that are included in the estimates relate to, for example, income^3^, population^3,27, 50^, road infrastructure^45^, transportation quantity^45^. For example, binary variables are also used to indicate countries that implemented carbon prices^51,52^.

Some studies on the CO_2_ emission impact of fuel taxes are focused on European data. For example, Zimmer and Koch examine the potential environmental effects of changes in tax rates applied to petrol and diesel oil in chosen EU member states, Norway and Switzerland^44^. Their results suggest that by adding a carbon tax (of a rate of 50 euros per tonne of CO_2_) to existing petrol and diesel taxes, CO_2_ emissions from road transportation in the analysed EU member states would be reduced by 7.2–10% (or 0.09–0.25 tonnes per capita) in 2020 compared to 2013. The authors also analyse the potential effects of the equalisation of diesel and petrol tax levels. Their findings show that the abolition of the favourable tax treatment of diesel oil would reduce CO_2_ emissions in some EU member states even more than the carbon tax.

Hájek et al. examine the effectiveness of environmental taxes and the EU emissions trading system for aviation to reduce transport-related greenhouse gas (GHG) emissions per capita in four groups of European countries: EU-27, old EU member states (i.e. EU-15 without Denmark, Finland, and Sweden), new EU member states and four Nordic countries (including Norway). A key explanatory variable used in their regression analysis is transport tax revenues per capita. The control variables used in the analysis include, inter alia, energy taxes used in transport (as % of GDP) and prices of EU aviation allowances. A negative partial impact of the former on GHG emissions has been found (at the 5% significance level) in the group of old EU member states^52^.

Transport taxes, energy taxes and other environmental taxes (expressed as a percentage of GDP) were also used as variables by Aydin and Esen, who investigated the impact of the taxes on CO_2_ emissions per capita in 15 EU member states^50^. Using data from 1995 to 2013, the authors have revealed that energy taxes reduce emissions if the energy tax revenue as % of GDP is higher than 2.2. Below this threshold, the result is not statistically significant.

Energy taxes are also found relatively ineffective in achieving climate policy objectives^53^. Using the Granger causality test, they found no statistically significant relationship between energy taxes per 1 toe of energy consumption and greenhouse emissions per capita in most EU-28 countries. They even found a positive relationship in one of the countries (Italy). The relationship between the two variables was negative only in three EU member states.

A relationship between energy taxes and CO_2_ emissions per GDP in the 27 EU member states (without Croatia) was investigated by Jeffrey and Perkins^54^. Their results imply that a 1% increase in the implicit tax rate on energy (energy tax revenues per 1 toe of consumed energy) causes a 0.11% decline in the so-called overall carbon intensity.

Morley (2012) employs panel data to examine the impact of environmental taxes (environmental tax revenue as a percentage of GDP and as a percentage of total tax revenue) on GHG emissions in EU member states and Norway^55^. He notes that both environmental taxes and emissions are negatively and significantly related.

There are also studies analysing energy taxes (along with other environmental taxes) in both European and non-European countries. Best et al. (2020) use a model of more than 100 countries to investigate the impact of carbon pricing and other policy instruments on both road and non-road CO_2_ emissions from fuel combustion^51,52^. They have found that the coefficient between carbon taxes and the average annual road CO_2_ emissions growth rate is negative and significant at 1%. Hence, the 1 euro per tonne CO_2_ increase in carbon taxes reduces 0.1 percentage points in the road sector's annual emissions growth rate. The result is similar regardless of whether the authors included the net petrol tax variable, estimated as a difference between the local retail price and an international price used as a benchmark.

Miller and Vela investigated the impact of energy and other environmental taxes (expressed as a percentage of GDP) on the growth rate of CO_2_ emissions per capita in the group of European and non-European countries. The study found a negative relationship between energy taxes and changes in emissions per capita^56^.

The research on the environmental effectiveness of energy taxes was also conducted in individual EU member states. For example, Runst and Höhle examined the effects of fuel tax increases in Germany between 1999 and 2003^45^. During this period, the tax rates applied to petrol and diesel increased by 15.35 cents^57^. The authors estimate that after 1999, implementing the “eco tax” decreased annual CO_2_ emissions per capita in Germany from transport by 0.2 to 0.35 t. The authors consider this effect considerable as there was a 9–18% reduction in transport emission levels in Germany in the 1990s^45^.

Frondel and Schubert estimated the long term effects of carbon prices introduced in Germany in 2021. They found that the carbon price on CO_2_ emissions from petrol and diesel oil (25 euros per tonne of CO_2_) will reduce emissions by 1.0 and 2.3 million tonnes, respectively^58^. The effects of “CO2-Preis” on carbon emissions were also investigated by Alberini et al.^43^. They found that the carbon price will reduce emissions from passenger cars by 1.0–1.4%.

A number of research studies focus on the impact of fuel taxes on carbon emissions in Sweden. As early as in 1998, Bohlin stated that the Swedish carbon tax “had no discernible effects in the transport (…) sector”^59^. In contrast, a more recent study found that the carbon tax levied on petrol in Sweden effectively reduced CO_2_ emissions^46^. Moreover, Andersson (2019) estimated that the Swedish carbon tax reduced CO_2_ emissions in the transport sector by 1.5 million tons (0.17 tons per capita) or by 6.3% in an average year from 1990 to 2005^27^. Together with the VAT on transport fuels, the carbon tax was supposed to reduce emissions from transport by almost 11%. According to Runst and Höhle, the results obtained by Andersson show that taxes in transport prove to have a considerable impact on reducing emissions^27,45^.

The results of research conducted in different countries and regions should be treated with caution as many country- or region-specific differences can impact transport CO_2_ emissions. It is crucial when comparing European and non-European countries. For example, the share of diesel in passenger car fuel consumption in Europe is much higher than in the US^27^. Other significant differences between European and non-European countries include the general level of motor fuel taxes^16^, the share of taxes in the price of fuels^18^, differences in taxation of petrol and oil^7^, the level of elasticity^12^, distances driven, , availability of alternative means of transportation or the magnitude of the rebound effect^60^.

Davis and Kilian estimated the impact of the proposal for an increase in the petrol tax rate in the US on carbon dioxide emissions. Their results suggest that the tax increase (by 10 cents per gallon of petrol) would reduce vehicle emissions by 1.5%^14^.

It is worth citing two studies based on counterfactual scenarios. According to Rivers and Schaufele, the carbon tax reduced petrol CO_2_ emissions by 2.4 million tonnes during the first four years^22^. Antweiler and Gulati found that the carbon tax in British Columbia affected both fuel demand and vehicle’s fuel efficiency. Compared to the situation without the tax fuel, demand per capita and average fuel efficiency of vehicles would be 7% higher and 4% lower, respectively^49^.

According to Kim et al., the hypothetical carbon tax in South Korea of a rate of about 54 U.S. dollars would reduce CO_2_ emissions by 916 thousand tonnes (about 1% of the transport emissions in Korea in 2007)^8^. The authors consider carbon reduction substantial. The results would be higher if the substitutability between petrol and diesel oil is included in the estimates.

Fiscal policy's effectiveness in reducing fuel consumption and CO_2_ emissions depends on the price elasticities of fuel demand^61^. There are many price (and income) elasticity estimates using different methodologies^61,62^. The long-term price elasticities of fuel demand are usually higher than the short-term ones but also less than unity^6^. Price elasticity estimates are often higher for petrol than diesel^36^. Price elasticity also differs across UE member states. Hence, tax rates necessary to achieve the EU reduction target (reduction of GHG emissions in transport by 20% in 2030 compared to 2008) should also differ from country to country^62^.

Some country-based studies indicate that transport fuel consumption is relatively inelastic to changes in price for diesel consumption in Portugal^36^

Studies suggest that consumers' demand response to fuel tax changes may be stronger than demand response to equivalent changes in tax-inclusive fuel prices^15,22^. Fuel tax changes may also be more salient to consumers than equal-sized changes in market prices of transportation fuels^22^. An explanation of this phenomenon is that tax rate changes are perceived to be more persistent than changes in other components of the fuel price^15^.

## Data

We studied 28 EU member states, including Croatia and the United Kingdom, for the period from 2010 to 2019. As of 1 January 2010, higher minimum levels of taxation applicable to diesel oil were in force in the EU, as set out in Annex I to Directive 2003/96/EC. In 2020 and beyond, the pandemic could impact car use. A decline in transport activities in 2020 resulted in a significant decrease in transport emissions.

Data on tax rates are taken mainly from the European Commission^31^, which publishes levels of taxation according to the article 4(2) of the 2003/96/EC Directive. It includes all indirect taxes except value-added taxes. We have excluded VAT as the standard rates that apply to fuels are similar in UE countries, and in the case of business consumers of fuels, VAT is primarily neutral. Data on tax rates collected from the European Commission were supplemented by other sources, including International Energy Agency.

The study is based on tax rates applicable to petrol and diesel oil used in road transport. We use tax rates applicable to fuels with a sulphur content of up to 10 mg/kg. Additionally, the rates for unleaded petrol with an octane number of 95 were considered in the case of petrol. The study uses tax rates effective as of 1 January, 2010 and subsequent years. Taxes on LPG and CNG were not considered due to the small share of these fuels in the total energy consumption in transport.

Data on CO_2_ emissions are collected from national inventory submissions 2021 published on the UNFCCC secretariat website^4^.

Many other factors directly or indirectly impact emissions, but many of them are difficult to quantify, and in many cases, international comparisons are impossible. Taxes and subsidies for car purchases and exploitation might be excellent examples. How to quantify the French system of bonus-malus applied to sales of new cars, which favours low emission cars at the expense of high emission, oversized and heavy cars? How to assess its impact on emissions? We would like to include this type of variables in our model, or at least some proxies, but that is impossible. Therefore, we use some economic indicators and other variables that might impact emissions along with fuel tax rates.

All the explanatory variables except fuel tax rates come from Eurostat or OECD databases.

The set of explanatory variables presented in Table 1 contains basic descriptive statistics about these variables for 2010–2019 in the group of analysed European countries. It can be noted that this group is relatively heterogeneous in terms of the studied statistics related to both petrol and diesel taxation, as well as other characteristics related to road transport, rail transport, road infrastructure and urbanisation. At the same time, what draws attention is the considerable need for observations in the dataset. Hence, in further analyses, we will look more at the relationships between the set of explanatory variables and their application in estimated equations.

**Table 1:** Presents the descriptive statistics of variables.

Explanatory variable	Obs	Mean	SD	Min	Max
Petrol tax (euro)	276	536.41	114.68	298.66	787.73
Diesel tax (euro)	276	421.92	91.80	245.00	703.76
GDP per capita (1000 euro)	280	27.86	18.83	5.80	97.85
Share of petrol cars with engines smaller than 2 L (%)	206	93.83	4.98	77.89	98.72
Share of diesel cars with engines smaller than 2 L (%)	204	78.17	11.44	39.13	92.11
Share of electric cars (%)	177	0.12	0.17	0.00	1.23
Share of cars heavier than 1500 kg (%)	152	28.96	21.78	1.99	91.41
Share of cars older than 10 years (%)	201	51.45	17.32	13.80	85.67
Rail passenger (m km-passenger per capita)	232	0.64	0.41	0.08	1.49
Urbanisation rate (%)	258	40.05	20.73	11.09	100.00
Population density (people/km^2^)	280	176.81	260.99	17.62	1595.13
Road density (km/km^2^)	248	1.49	1.58	0.11	9.03
Motorways density (km/km^2^)	259	0.02	0.02	0.00	0.08
Vehicles passenger (m km-passenger per capita)	154	8.66	4.43	0.59	14.64

Source: own calculations based on European Commission, Eurostat and OECD, 2022 data.

## Methodology, results and discussion

In order to estimate the impact of fuel taxes on CO_2_ emissions, we constructed equations, the first of which, the explanatory variable, is CO_2_ emissions to a country’s GDP. In the second equation, we estimate the parameters of the equation in which the explanatory variable is per capita emissions. In addition to the tax variables, the control variables include data on the level of socio-economic development, road infrastructure, and the characteristic of the vehicles fleet.

As the first step, however, we look at the correlations between the explanatory variables. We present estimated pairwise correlation coefficients in Table 2.

**Table 2:** Estimated pairwise correlation coefficients.

	Petrol tax	Diesel tax	GDP per capita	Petrol cars with engines up to 2 l	Diesel cars with engines up to 2 l	Share of electric cars	Share of cars above 1500 kg	Share of cars older than 10 years	Rail passenger km per capita	Urbanisation rate	Population density	Road density	Motorways density
Diesel tax	0.7551*	1											
	304	304											
GDP per capita	0.3837*	0.2796*	1										
	303	303	474										
Petrol cars with engines up to 2 l	− 0.1071	− 0.0416	− 0.5575*	1									
	202	202	291	291									
Diesel cars with engines up to 2 l	0.0957	0.0553	− 0.0777	0.4857*	1								
	200	200	289	289	289								
Share of electric cars	0.3381*	0.1589*	0.3594*	− 0.1623*	0.0015	1							
	176	176	177	154	152	177							
Share of cars above 1500 kg	0.2499*	0.2475*	0.2091*	− 0.2454*	− 0.0482	− 0.062	1						
	148	148	214	192	191	114	214						
Share of cars older than 10 years	− 0.4926*	− 0.4060*	− 0.7004*	0.1841*	− 0.1477*	− 0.2876*	0.1042	1					
	198	198	309	260	258	139	198	309					
Rail passenger km per capita	0.4242*	0.4654*	0.4969*	− 0.1283*	0.0824	0.3828*	0.0201	− 0.6085*	1				
	249	249	379	234	232	141	167	248	379				
Urbanisation rate	0.3839*	0.3384*	0.2542*	− 0.2157*	− 0.0058	0.1859*	0.3141*	− 0.1200*	0.2151*	1			
	280	280	412	265	263	161	188	283	361	413			
Population density	0.1517*	0.1403*	0.0378	0.1555*	0.2566*	0.1153	0.0996	− 0.0496	0.3784*	0.7094*	1		
	304	304	474	291	289	177	214	309	379	413	475		
Road density	0.1329*	0.088	0.0705	0.0902	0.1563*	0.1728*	− 0.0048	− 0.0438	0.3035*	0.5477*	0.9040*	1	
	244	244	374	266	264	161	207	279	324	343	374	374	
Motorways density	0.3995*	0.0722	0.5231*	− 0.045	0.1546*	0.3974*	− 0.099	− 0.5020*	0.2666*	0.4182*	0.8224*	0.5691*	1
	255	255	311	230	228	168	167	234	268	284	311	288	311
Vehicles passenger km per capita	0.7112*	0.5395*	0.7277*	− 0.5111*	− 0.3571*	0.2691*	0.3749*	− 0.2696*	0.5409*	0.2810*	0.2319*	0.3896*	0.3975*
	150	150	226	146	144	86	112	160	217	223	226	214	183

*Denote statistical significance at a 5% level.

No of observations under estimated coefficients.

*Source*: own calculations.

The correlation table above shows that the dataset is a highly unbalanced panel. In particular, if we look at the number of observations in the first two columns with correlation results for the petrol and diesel taxes, it can be noted that in many cases, the number of observations in individual pairs is less than 200. Analysing the coefficients of linear correlation between the explanatory variables, it could be revealed that some variables are strongly correlated. In particular, it concerns the density of roads and population (corr 0.9), as well as the density of motorways and population density (corr 0.82). Car and rail passenger movement are strongly correlated (corr 0.54), and the former quite strongly with GDP per capita (corr 0.73). Wealthier economies with higher GDP per capita seem to have a higher proportion of electric cars, a better developed motorway network and a lower share of older cars. Countries with a higher fraction of older cars have worse passenger rail traffic (corr 0.61). The proportion of cars with smaller petrol and diesel engines negatively correlates with lower vehicle passenger traffic (corr − 0.51 and − 0.36). At the same time, smaller petrol-engined cars are more prevalent in poorer countries (corr − 0.56). Given the limitations on the number of observations in each pair mentioned above, as well as correlations between explanatory variables indicating potential sources of collinearity, we decided to use the following:

1. Petrol tax – the tax on petrol
2. Diesel tax – the tax on diesel
3. GDP per capita
4. Urbanisation rate
5. Population density
6. Dummy variable for a carbon tax. The dummy equals 1 if a country introduced a carbon tax on petrol and diesel oil in a given year.

We restricted our analyses to 26 UE countries because we excluded Cyprus due to the lack of data and Luxemburg since in this country, petrol is cheaper than in the neighbouring countries. Thanks to foreigners’ purchases, the data on emissions could be more reliable.

Using road and car CO_2_ emissions data and a set of explanatory variables, the equation parameters were estimated using panel methods, namely linear regression with panel-corrected standard errors. Estimate standard errors are panel corrected for panel-level heteroscedasticity, so we allow that each country has a different variance of the disturbances. As we agree to autocorrelation within panels, there is a first-order autocorrelation process^63^.

CO_2_ emissions variables and taxes are expressed in logarithms; thus, the estimates can be interpreted as elasticities.

The figures below present the estimated parameters related to the impact of petrol and diesel tax rates on CO_2_ emissions. We distinguish different types of emissions: road, diesel road, petrol road, car, diesel car and petrol car emissions.

**Figure 6:**
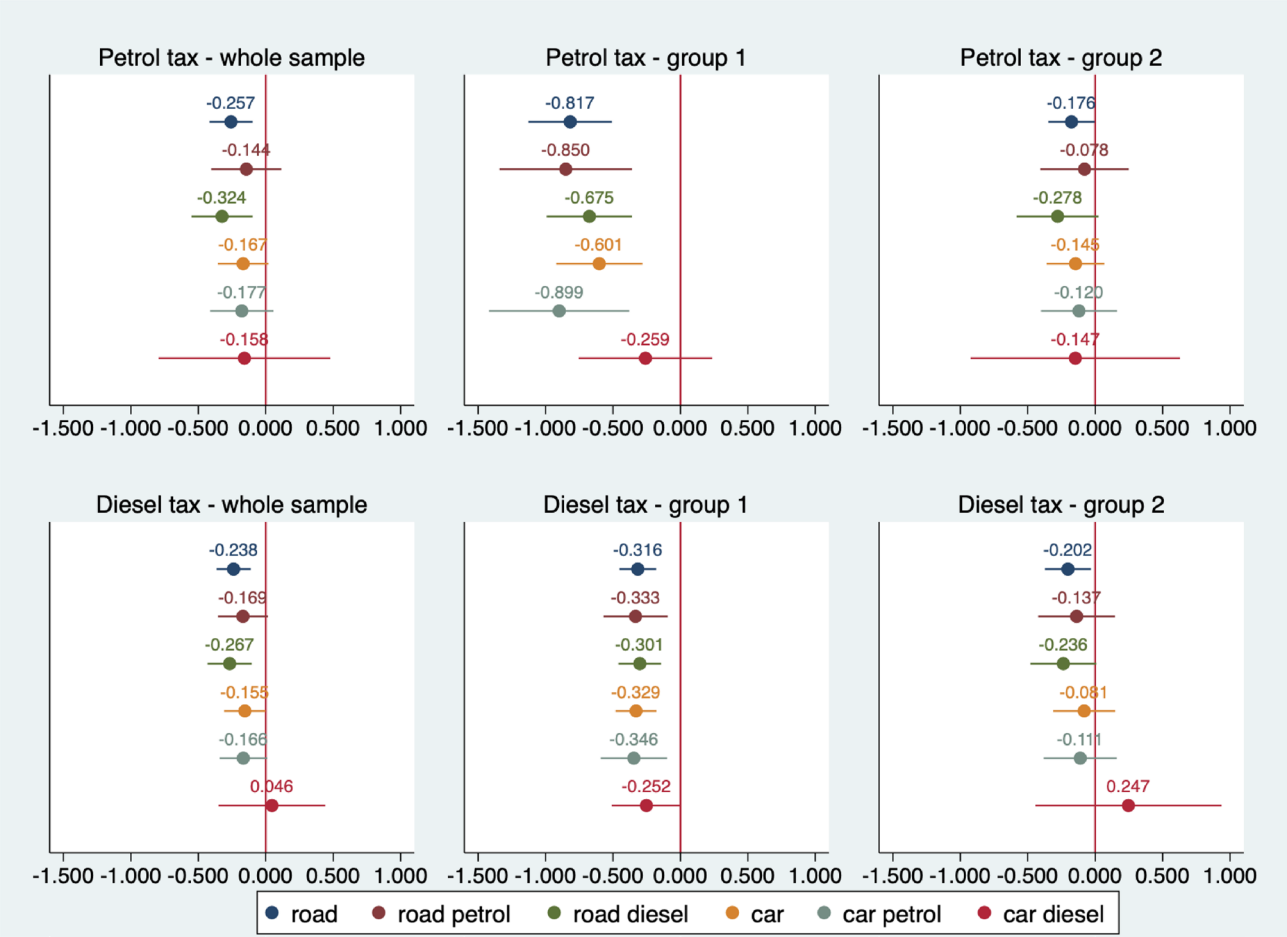
Estimated elasticities of CO_2_ emissions per GDP due to petrol and diesel tax rates (point estimates) with a 95% confidence interval. Group 1 includes Belgium, Denmark, Finland, France, Germany, Ireland, Italy, Netherlands, Sweden and the United Kingdom. Group 2 includes Austria, Bulgaria, Croatia, the Czech Republic, Estonia, Greece, Hungary, Latvia, Lithuania, Malta, Poland, Portugal, Romania, Slovakia, Slovenia, and Spain. *Source*: own calculations

**Figure 7:**
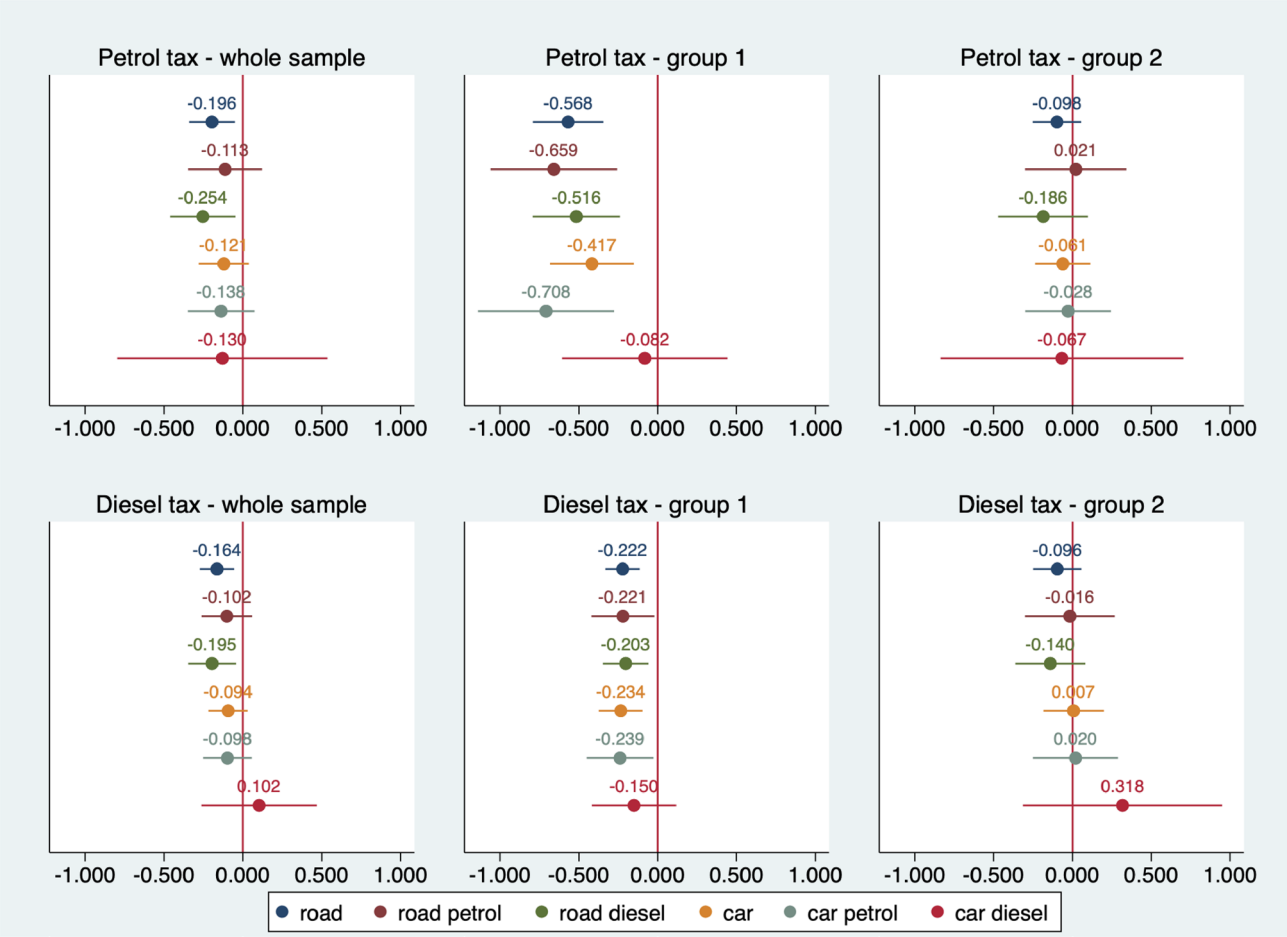
Estimated elasticities of CO_2_ emissions per capita due to petrol and diesel tax rates (point estimates) with a 95% confidence interval. Group 1 and 2, as in Fig. 6. *Source*: own calculations.

The equation parameters were estimated in several variants. Firstly, we considered the whole sample. In Fig. 6, emissions (6 types) are expressed in relation to GDP, while Fig. 7 shows per capita emissions. Group 1 comprises countries with higher fuel taxes, and Group 2 consists of countries with lower tax rates. Each point on the graphs should be interpreted as an estimated elasticity of a given type of CO_2_ emissions concerning petrol tax rates (top panel) or diesel tax rates (bottom panel).

The estimates presented in Fig. 6 suggest that the effect of the petrol tax on CO_2_ emissions across the sample is relatively low. This effect is noticeable in the countries of the higher rate group, and higher (approx. − 0.8 to 0.9) for road, road petrol and petrol car emissions. It is generally lower for cars and road diesel emissions (0.6–0.7). At the 5% significance level, we found no statistically significant relationship between emissions and petrol tax in countries with low tax rates. The exception is total road emissions, with an estimated coefficient of − 0.176.

For the estimates of the impact of the diesel tax on CO_2_ emissions in the different cross sections, we see that the impact in the whole sample is similar to that observed for the petrol tax. The association between total road emissions (− 0.238) and diesel road (− 0.267) is statistically significant. The impact is observable in Group 1, but the estimated parameters are about two to three times lower than for petrol tax. At the same time, the estimated parameters in this group are less differentiated by the type of emissions (ca. − 0.30 to 0.35). The impact of the diesel tax on the different types of emissions in Group 2 is similar to what we found for the petrol tax impact.

A sensitivity analysis of the influence of the diesel and petrol tax on CO_2_ emissions was performed using a different measure of emissions, that is, converting them to per capita. As in Fig. 6, in Fig. 7, we also present parameter estimates of the impact of diesel and petrol taxes on per capita emissions for the whole sample and the two groups previously identified. The conclusions for the estimates are nearly the same: we see a statistically significant relationship between diesel and petrol tax and emissions primarily in Group 2, and the estimates for per capita emissions are about 0.1–0.2 lower than for the corresponding estimates in the emissions-to-GDP equation. The conclusions for the impact of taxes on emissions to GDP and per capita are similar. However, the clear statistical non-significance of the estimates in the 2nd group of countries should be pointed out. This confirms previous findings that fuel taxes have to be high to influence emissions.

Our findings are in line with those studies which found a negative relationship between energy or environmental taxes and carbon dioxide emissions^55,56^. The impact is somewhat limited; however, it is stronger in countries with higher fuel taxes, including countries with higher carbon taxes like Sweden. Other studies also indicate that the environmental effectiveness of 'green' taxes differs between EU member states^52^.

## Conclusion and policy implications

In the 2010–2019 years, the taxation of petrol and diesel oil increased in many EU member states. In the case of some countries, this rise resulted from the introduction of carbon taxes. The rate changes were discretionary and were usually dictated by the need for an increase in budget revenues and the willingness to limit CO_2_ emissions and other negative external effects connected to car transport. Apart from Belgium, the equalisation of diesel taxation and petrol taxation did not succeed despite the shared awareness of higher emissions of harmful substances from diesel engines. The minimal rates resulting from the directive on minimal tax rates did not change either. Unlike in the case of cigarettes (a robust increase of minimal taxation) and similarly to the case of alcohol, the EU could not agree on increasing the minimum rates and equalising the minimal rate for petrol and oil.

Based on previous research, we analysed 26 EU member states over 10 years that ended just before the pandemic. We focused on passenger cars, and the critical question was whether the newer data confirmed the previous findings. Newer data encompassed more countries and a longer period than most previous research studies. Moreover, in the second decade of this century, the social awareness of climate change was definitely much more prominent than when most other analyses were conducted.

There are many factors influencing emissions of CO_2_, i.e., economic activity, fuel efficiency, the number and the structure of vehicles, availability of public transportation and cost of purchasing and using a passenger car. Governments can influence to a certain degree, most of these factors via taxation, subsidies and command and control policies. Taxation of fuels is the oldest instrument influencing emissions, even though it was introduced to bring money, not to internalise the negative externalities. Nowadays, not losing their revenue-yielding function, taxes on fuels are also used to curb emissions and other externalities. It is challenging to analyse the pure impact of fuel taxes on emissions as taxes are accompanied by other, usually country-specific measures such as subsidies to low/zero emission cars, differentiated taxation of purchase and possession of the car and the like.

Our research confirms previous findings that fuel taxes have a moderate impact on general road emissions and, in some cases, also on passenger car emissions. This holds no matter if we relate fuel taxes to emissions per GDP or per capita. We estimated the elasticities of the influence of fuel taxes on CO2 emissions. These elasticities tend to be bigger and more significant in countries with higher taxes. This might be an essential argument in a political debate, especially nowadays, when high energy prices due to Russian aggression in Ukraine increase political pressure to lower fuel taxes. It is a very bad policy. First, a decrease in the tax rate is an easy task; a return to the previous level is far more complicated. Second, in the case of transportation fuels, the price increases were not that big (compared to natural gas or electricity), and the best incentive to reduce demand (and therefore indirectly price) is to keep the price relatively high. Third, this boosts the 'Santa Claus mentality,' i.e., the perception that government can give a gift to society at no cost.

Taxes on fuels are specific taxes; therefore, the real burden falls with inflation and growing earnings, increasing fuel's economic affordability and demand for them. Thus, they should be adjusted over time to send a correct and strong signal. This is not easy from a political perspective, however. In the public discussion, the authorities should stress the growing economic affordability of fuels and the negative consequences of car transportation. The awareness that (high) fuel taxes can play a quite significant role in reducing demand for fuels and therefore reducing dependence on imported fuels and emissions as well other negative externalities, might reduce social resistance against higher fuel taxes.

## Data Availability

The data that support the findings of this study are available from the corresponding author on request.
